# Adipose-derived stem cells therapy effectively attenuates PM_2.5_-induced lung injury

**DOI:** 10.1186/s13287-021-02441-3

**Published:** 2021-06-19

**Authors:** Junling Gao, Juntao Yuan, Qun Liu, Yuanli Wang, Huiwen Wang, Yingjie Chen, Wenjun Ding, Guangju Ji, Zhongbing Lu

**Affiliations:** 1 College of Life Science, University of Chinese Academy of Sciences, 19A Yuquanlu, Beijing, 100049 China; 2 Institute of Biophysics, Chinese Academy of Sciences, Datun Road 15, Chaoyang district, Beijing, 100101 China; 3 Department of Physiology and Biophysics, University of Mississippi Medical Center, Jackson, USA

**Keywords:** Adipose-derived stem cells, PM2.5, inflammation, Pyroptosis

## Abstract

**Background:**

The adverse health effects of fine particulate matter (PM_2.5_) exposure are associated with marked inflammatory responses. Adipose-derived stem cells (ADSCs) have immunosuppressive effects, and ADSC transplantation could attenuate pulmonary fibrosis in different animal disease models. However, whether ADSCs affect PM_2.5_-induced lung injury has not been investigated.

**Method:**

C57BL/6 mice were exposed to PM_2.5_ every other day via intratracheal instillation for 4 weeks. After that, the mice received tail vein injections of ADSCs every 2 weeks.

**Results:**

ADSC transplantation significantly attenuated systemic and pulmonary inflammation, cardiac dysfunction, fibrosis, and cell death in PM_2.5_-exposed mice. RNA-sequencing results and bioinformatic analysis suggested that the downregulated differentially expressed genes (DEGs) were mainly enriched in inflammatory and immune pathways. Moreover, ADSC transplantation attenuated PM_2.5_-induced cell apoptosis and pyroptosis in the lungs and hearts.

**Conclusion:**

ADSCs protect against PM_2.5_-induced adverse health effects through attenuating pulmonary inflammation and cell death. Our findings suggest that ADSC transplantation may be a potential therapeutic approach for severe air pollution-associated diseases.

**Supplementary Information:**

The online version contains supplementary material available at 10.1186/s13287-021-02441-3.

## Introduction

Increased airborne fine particulate matter (PM_2.5_, aerodynamic diameter ≤ 2.5 μm) concentrations (higher than the World Health Organization air quality guideline level — 10 μg/m^3^) have been implicated in the development of respiratory and cardiovascular diseases, including chronic obstructive pulmonary disease (COPD) [1], asthma [2], bronchitis [3], coronary artery disease [4], and atherosclerosis and congestive heart failure [5, 6]. Due to its small size, PM_2.5_ is easily inhaled into the airway and largely deposited in lung alveoli. Although the mechanism underlying the cytotoxicity of PM_2.5_ remains elusive, dysregulation of the inflammatory response is considered a major trigger for cell apoptosis and lung injury [7, 8]. Thus, strategies with anti-inflammatory effects might be effective in attenuating PM_2.5_-induced disease [9].

It is well documented that adipose-derived mesenchymal stem cells (ADSCs) have multilineage differentiation potential and can differentiate into different cell types, including adipocytes, osteoblasts, chondrocytes, and hepatocytes [10]. ADSCs can also repair damaged organs and tissues through releasing a variety of paracrine factors and extracellular vesicles (EVs) [11]. These properties render ADSCs attractive therapeutic agents for tissue defects and degenerative diseases. Interestingly, ADSCs have the ability to differentiate into type 2 alveolar epithelial cells [12], and ADSC therapy ameliorates lung injury and fibrosis in a series of pulmonary disorders, including bronchiolitis obliterans [13], idiopathic pulmonary fibrosis [14], and emphysema [12]. Moreover, EVs derived from ADSCs could also alleviate PM_2.5_-induced lung injury in mice [15]. However, whether ADSCs exert a direct protective effect on PM_2.5_-induced lung injury remains unclear.

## Materials and methods

### Reagents

Enzyme-linked immunosorbent assay (ELISA) kits for interleukin 6 (IL-6), IL-1β, tumor necrosis factor alpha (TNFα), and 3′-nitrotyrosine (3′-NT) were purchased from Abcam PLC (#ab100712, #ab197742, #ab108910, and #ab116691, Cambridge, UK). 4-Hydroxynonenal (4-HNE) ELISA kit was obtained from Donggeboye Biological Technology Co. Ltd. (#DG30947M, Beijing, China). TUNEL staining kits were obtained from the Beyotime Institute of Biotechnology (#C1088 or #C1090, Shanghai, China). Dulbecco’s modified Eagle medium/nutrient mixture F-12 (DMEM/F12), fetal bovine serum (FBS), and TRIzol were obtained from Thermo Fisher Scientific Inc. (#11320033, #10099141, #15596026, Waltham, MA, USA). PM_2.5_ was collected using high-volume sampler particle collectors and the morphology, size distribution, and components of the constituents were described in a previous study [16]. All other chemicals made in China were of analytical grade.

### ADSCs isolation and characterization

White adipose tissues were harvested from male C57BL/6J mice at the age of 6–8 weeks. After washing two times with ice-cold phosphate buffered saline (PBS), the adipose tissue was mechanically diced, digested, washed, filtered, and centrifuged. The cell pellet was washed with DMEM/F12 one more time and then resuspended in DMEM/F12 supplemented with 10% FBS, and 1% penicillin and streptomycin. Finally, ADSCs were placed in a 10-cm dish and cultured in an incubator at 37 °C with 5% CO_2_.

To confirm that ADSCs were successfully isolated from white adipose tissue, the expression of cell surface markers of ADSCs were evaluated by flow cytometry. FITC-conjugated CD44, eFluor® 450-conjugated CD34, and PE-conjugated CD73 and CD90.2 antibodies were purchased from Thermo Fisher Scientific Inc. (#11-0441-81, #48-0341-80, #12-0731-81, and #12-0903-81). The cells were incubated with an antibody against each of the cell surface markers for 30 min and then subjected to flow cytometry analysis.

To induce differentiation, ADSCs were cultured in adipogenic differentiation medium (DMEM/F12, 10% FBS, 1 μM insulin, 0.5 μM dexamethasone, 0.5 mM 3-isobutyl-1-methylxanthine, and 50 μM indomethacin) or osteogenesis differentiation medium (DMEM/F12, 10% FBS, 5 μg/ml ascorbic acid, 10 nM dexamethasone, 10 mM β-glycerophosphate, 10 nM 1α, 25-dihydroxyvitamin D3) for 14 days. After differentiation, the cells were fixed with 10% formalin, washed, and then stained with Oil red O or alizarin red solution.

### Experimental animals

Twenty-four 8-week-old male C57BL/6J mice were randomly divided into the control group (8 mice) and PM_2.5_-exposed group (16 mice). The mice were first treated with 10 μl PBS (control group) or 10 mg/kg PM_2.5_ in 10 μl PBS (PM_2.5_-exposed group) every other day via intratracheal instillation for 4 weeks. Based on a previous report [17], the dose used here was equal to daily exposure to ~ 1500 μg/m^3^ PM_2.5_ for 1 month. After PM_2.5_ exposure, mice were randomly assigned to 2 groups, 1 group (8 mice) received ADSCs (2 × 10^5^ cells/mouse) via tail vein injection at the fourth and sixth weeks and the other treated with PBS. At the end of the eighth week, the mice were sacrificed by CO_2_ inhalation followed by cervical dislocation. To avoid the errors arising from bias, the group information was blind to the researchers who performed biochemical assays and histological analysis. During the whole experimental period, the mice were housed in cages with corn cob bedding, and were fed commercial mouse chow and distilled water ad libitum. The mice were housed under controlled temperature (22 ± 2 °C) and relative humidity (40–60%) conditions with a 12-h light/dark cycle. Mice with any abnormalities in skin, hair, bodyweight, and behavior were dropped out from the group.

### Histopathological staining

After perfusion with PBS, the mouse lungs were harvested, washed, fixed with formalin, and embedded in paraffin. Lung sections (5 μm) were stained with hematoxylin and eosin (H&E) stain, a Masson’s trichrome stain kit (#G1340, Solarbio Science & Technology Co. LTD, Beijing, China), dihydroethidium (DHE), or a TUNEL staining kit. The neutrophil/Ly-6B is a heavily glycosylated protein expressed on neutrophils [18], while galectin-3 (Gal-3, also called Mac-2 antigen) is a carbohydrate-binding protein expressed on the surface of inflammatory macrophages [19]. To assess neutrophil and macrophage infiltration, lung sections were also stained with Gal-3 and neutrophil antibodies, respectively. Five mice per group were used for these experiments.

### ELISA measurement

Serum TNFα, IL-1β, and IL-6 levels and pulmonary 3′-NT and 4-HNE levels were determined using commercial ELISA kits according to the manufacturer’s instructions. In brief, samples or standard plus antibody cocktail were added to the appropriate wells of the pre-coated microplate. The plate was then incubated overnight at 4 °C with gentle shaking. After washing and adding the chromogen substrates for 10 min, the reactions were terminated by adding the stop solution. The content was calculated according to the standard curve and OD value.

### Echocardiographic measurement

After anesthetization with 1.5% isoflurane, the mice were subjected to echocardiographic measurement using a VisualSonics high-resolution Veve 2100 system (Visual Sonics, Toronto, ON, Canada).

### RNA isolation and RNA-sequencing

Total RNA was extracted from the lungs (3 mice per group) using TRIzol reagent and RNA quality was measured with an Agilent 2100 bioanalyzer (Thermo Fisher Scientific, MA, USA). The isolated RNA was further purified by DNase I treatment and rRNA removal. Library construction and RNA-sequencing were then performed on a BGISEQ500 platform (BGI-Shenzhen, China).

### Read mapping and differentially expressed gene analysis

As described previously [20], the raw data were cleaned with SOAPnuke and trimmomatic software [21], and then the clean reads were mapped to the reference genome (Mus_musculus, GCF_000001635.25_GRCm38.p5) using HISAT [22] or Bowtie 2 [23] software. RESM software [24] was used to obtain the gene expression level and the differential expression of genes (DEGs) between two groups was assessed by DEGseq [25] with the following thresholds: fold change ≥ 2 and adjusted *P* value ≤ 0.001. The identified DEGs for each pair were mapped to terms in the Kyoto Encyclopedia of Genes and Genomes (KEGG) database (http://www.genome.jp/kegg/pathway.html). The *p* value was adjusted for the false discovery rate (FDR) to obtain the q-value, and a q-value ≤ 0.05 was considered to indicate significant enrichment.

Gene set enrichment analysis (GSEA) was also performed with the KOBAS 3.0 online tool 1 [26] and Java GSEA2 [27]. An FDR q-value ≤ 0.25 was considered to indicate significant enrichment.

### Quantitative real-time PCR and western blotting

A PrimeScript RT reagent kit (#RR036B, TaKaRa, Otsu, Japan) was used for cDNA synthesis. A quantitative real-time polymerase chain reaction (qPCR) assay was performed using the SYBR Premix Ex Taq™ II Kit (#RR820DS, TaKaRa) and the results were normalized to the level of 18 S ribosomal RNA. The primers used in the qPCR assay are listed in Table S1.

Proteins were extracted from the lungs using buffer (50 mM Tris-Cl, 150 mM NaCl, 100 μg/ml phenylmethylsulfonyl fluoride, protease and phosphatase inhibitor cocktail (#046931124001 and #4906837001, Roche, Basel, Switzerland), and 1% Triton X-100) on ice for 30 min. After centrifugation at 12000×*g* at 4 °C for 20 min, the supernatant was used for western blot analysis. The detail information for antibodies are listed in Table S2.

### Data and statistical analysis

All values are expressed as the mean ± standard deviation (SD). Normal distribution was assessed by the Kolmogorov-Smirnov normality test. The significance of differences was tested using one-way analysis of variance (ANOVA) followed by Fisher’s least significant difference test or the Kruskal-Wallis nonparametric test followed by Dunn’s test using GraphPad Prism 7 (GraphPad Software Inc., CA, USA). Statistical significance was defined as *p* < 0.05.

## Results

### Characterization of ADSCs and in vitro differentiation assay

As shown in Figure S1, the isolated ADSCs were positive for CD44 (93.5%), CD73 (97%), and CD90 (95.8%) and negative for CD34 (3.49%). Microscopic observation showed that the cultured ADSCs exhibited a fibroblast-like appearance and formed a monolayer (Figure S2A). To determine the differentiation potential, ADSCs were cultured in adipogenic or osteogenic differentiation induction medium for 14 days. Positive staining with Oil red O and alizarin red indicated that ADSCs could differentiate into adipocytes and osteocytes, respectively (Figure S2B-C).

### ADSC transplantation attenuates PM_2.5_-induced inflammation and pulmonary fibrosis in mice

**Fig. 1:**
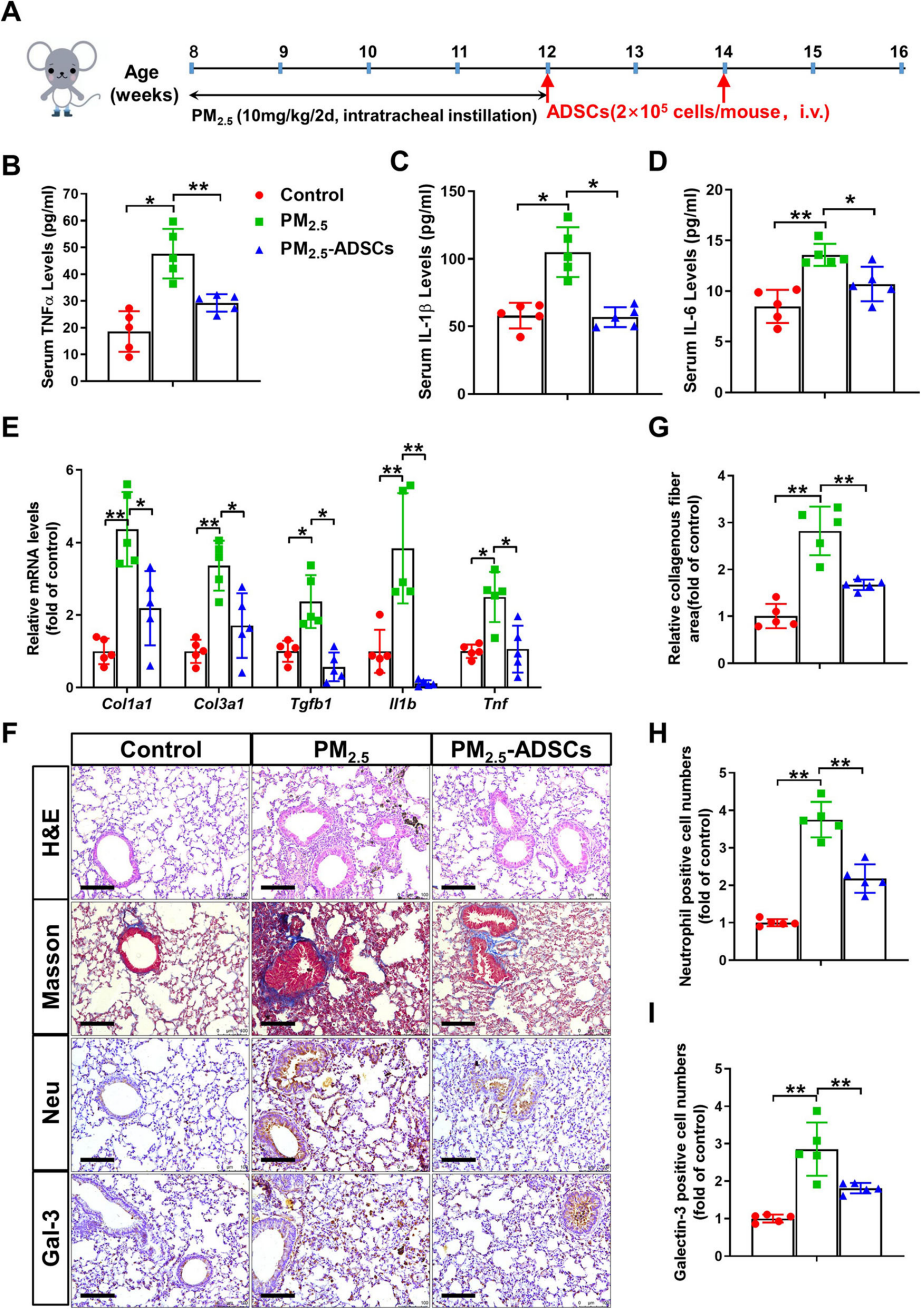
Adipose-derived stem cell transplantation alleviated PM_2.5_-induced inflammation and pulmonary fibrosis. C57BL/6 mice were administered PBS or 10 mg/kg PM_2.5_ every other day via intratracheal instillation for 4 weeks. At the fourth and sixth weeks, some of the PM_2.5_-exposed mice were treated with adipose-derived stem cells (ADSCs) via tail vein injection (2 × 10^5^ cells/mouse). **A** Schema of the experimental procedure. **B**–**E** After mice were sacrificed, serum TNFα (**B**), IL-1β (**C**), and IL-6 (**D**) levels and the mRNA levels of pulmonary inflammatory and fibrotic genes (**E**) were measured. **F** Representative lung sections were stained with hematoxylin and eosin (H&E) stain, Masson’s trichrome stain, and antibodies specific for neutrophils and macrophages (galectin-3, Gal-3) (brown staining). Scale bar = 100 μm. **G**–**I** The relative collagenous fiber area (**G**) and the numbers of neutrophils (**H**) and Gal-3-positive cells (**I**) were quantified. *N* = 5; the data are presented as the mean ± SD; asterisk indicates *p* < 0.05; double asterisks indicate *p* < 0.01

To investigate the protective effects of ADSCs on PM_2.5_-induced lung injury and cardiac dysfunction, we treated the PM_2.5_-exposed mice with ADSCs twice via tail vein injection. The processes of the ADSC transplantation experiment are illustrated in Fig. 1A. At the end of the experimental period, there were significant increases in serum TNFα, IL-1β, and IL-6 levels in PM_2.5_-exposed mice, while these increases were diminished in the mice that received ADSC transplantation (Fig. 1B–D). Next, we performed qPCR to examine the effects of ADSCs treatment on the mRNA levels of genes related to inflammation and fibrosis. PM_2.5_ exposure caused significant increases in pulmonary collagen I and III, transforming growth factor beta (TGF-β), IL-1β, and TNFα mRNA levels; however, these increases were significantly attenuated by ADSC transplantation (Fig. 1E). Histopathological analysis of lung sections using H&E and Masson’s staining demonstrated that the collapse of alveoli, thickening of airway epithelium, and deposition of collagen in the lungs of PM_2.5_-exposed mice were attenuated by ADSC treatment (Fig. 1F, G). Immunohistochemical staining with neutrophil and Gal-3 antibodies also showed that PM_2.5_-induced infiltration of neutrophils and macrophages in the lungs was diminished by ADSC transplantation (Fig. 1F, H, I).

### ADSC transplantation prevents PM_2.5_-induced pulmonary oxidative stress and cell apoptosis in mice

**Fig. 2:**
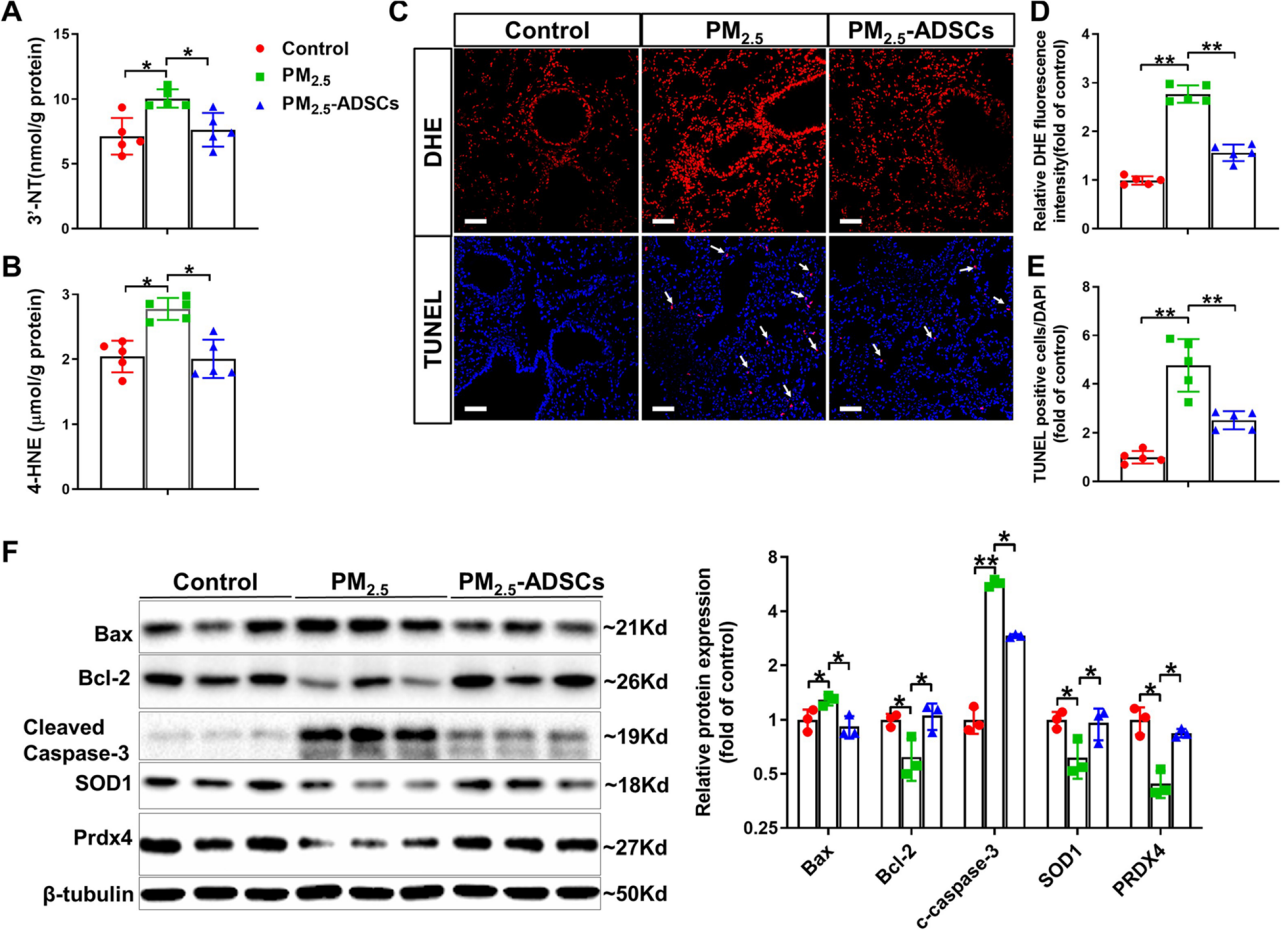
ADSC transplantation attenuated PM_2.5_-induced pulmonary oxidative stress and cell apoptosis. **A**, **B** After PM_2.5_ exposure and ADSC transplantation, the levels of 3′-nitrotyrosine (3′-NT) (**A**) and 4-hydroxynonenal (4-HNE) (**B**) in lung tissue were measured. **C**–**E** Lung sections from the control and PM_2.5_-exposed mice were stained with dihydroethidium (DHE) or DAPI (blue) and dye (red) from a TUNEL assay kit (scale bar = 50 μm, the arrows point to TUNEL-positive cells). The relative DHE fluorescence intensity (**D**) and TUNEL-positive cells (**E**) were quantified. **F** Lysates of lung tissue were subjected to western blotting to determine the expression levels of Bax, Bcl-2, cleaved caspase-3, superoxide dismutase 1 (SOD1), and peroxiredoxin 4 (PRDX4). β-Tubulin was used as a loading control. In **A**–**E**, *N* = 5; in **F**, *N* = 3; the data are presented as the mean ± SD; asterisk indicates *p* < .05; double asterisks indicate *p* < 0.01

PM_2.5_ exposure significantly increased pulmonary 3′-NT and 4-HNE levels, while these increases in the levels of oxidative stress markers [28] were attenuated by ADSC transplantation (Fig. 2A, B). Moreover, DHE staining revealed that ADSC decreased superoxide levels in PM_2.5_-exposed lungs (Fig. 2C, D), indicating that ADSC treatment ameliorated PM_2.5_-induced pulmonary oxidative stress. TUNEL staining showed that ADSC transplantation significantly attenuated the increases in apoptotic cell number in the lungs of PM_2.5_-exposed mice (Fig. 2C, E). Consistently, the expression of the proapoptotic protein Bax and cleaved caspase-3 was increased, whereas the expression of antiapoptotic protein Bcl-2 was decreased in the lungs of PM_2.5_-exposed mice. Moreover, PM_2.5_ exposure also decreased superoxide dismutase 1 (SOD1) and peroxiredoxin 4 (PRDX4) protein expression in the lungs. However, the changes in apoptosis and antioxidant related proteins were significantly attenuated by ADSC transplantation (Fig. 2F).

### ADSC transplantation has profound effects on the gene expression profile in PM_2.5_-exposed lungs

**Fig. 3:**
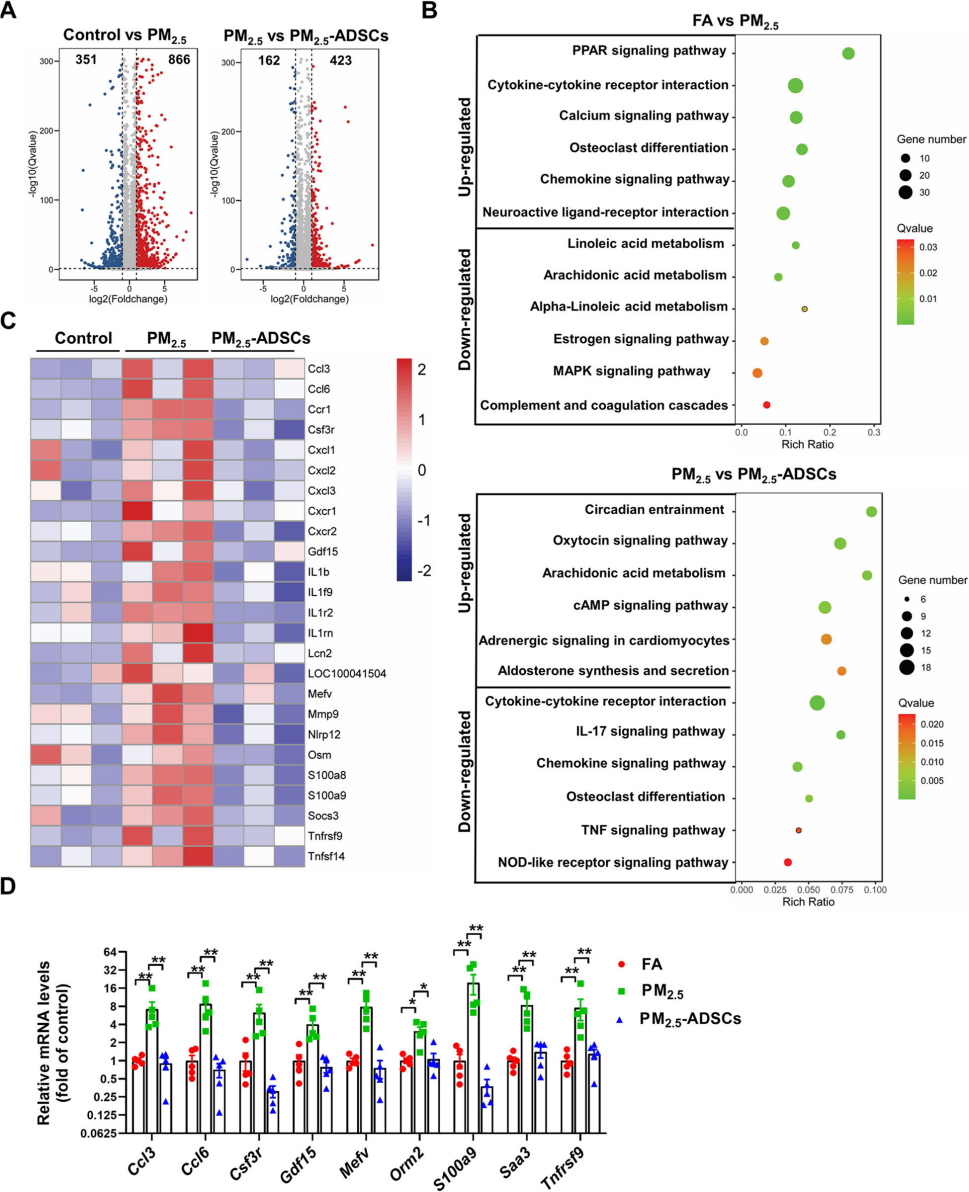
ADSC transplantation affected the gene expression profile in PM_2.5_-exposed lungs. **A** The fold changes in the expression of differentially expressed genes (DEGs) in the control group vs PM_2.5_ group and the PM_2.5_ group vs PM2.5-ADSC group were visualized in a volcano plot. **B** The advanced bubble chart shows the top 6 significantly enriched KEGG pathways of the up- and downregulated DEGs in the control group vs PM_2.5_ group and the PM_2.5_ group vs PM_2.5_-ADSC group. **C** The gene expression profiles of the downregulated DEGs in the PM_2.5_ group vs PM_2.5_-ADSC group that were enriched in inflammatory pathways, as determined by KEGG pathway analysis, are shown in the heat map. **D** To verify the RNA-sequencing results, mRNA levels were measured by real-time qPCR. In **A**–**C**, *N* = 3; in **D**, *N* = 5; the data are presented as the mean ± SD; asterisk indicates *p* < 0.05; double asterisks indicate *p* < 0.01

To investigate the molecular mechanism by which ADSC transplantation attenuated PM_2.5_-induced lung injury in mice, RNA-sequencing was performed to analysis the whole-genome expression profiling changes in these groups. We identified 1217 differential DEGs (866 upregulated and 351 downregulated) in the control group vs PM_2.5_ group and 585 DEGs (423 upregulated and 162 downregulated) in the PM_2.5_ vs PM_2.5_-ADSC group, and the fold changes of these DEGs were visualized in volcano plots (Fig. 3A). KEGG pathway enrichment analysis demonstrated that the upregulated DEGs in the control group vs PM_2.5_ group were mainly significantly enriched in inflammatory and metabolic pathways, including the PPAR signaling pathway, cytokine-cytokine receptor interaction, chemokine signaling pathway, and adipocytokine signaling pathway (Fig. 3B). Notably, the downregulated DEGs in PM_2.5_ group vs PM_2.5_-ADSC group were significantly enriched in inflammation and immune pathways, including the cytokine-cytokine receptor interaction, IL-17, chemokine signaling, TNF signaling, and NOD-like receptor signaling pathways (Fig. 3B). To explore the immunosuppressive mechanism of ADSCs in PM_2.5_-exposed lungs, the expression of the DEGs was visualized in a heatmap (Fig. 3C), which demonstrated that the expression of most of the inflammation-related genes that were upregulated in PM_2.5_-exposed lungs was repressed by ADSC transplantation. The qPCR results demonstrated that ADSC treatment attenuated the upregulation of *Ccl3*, *Ccl6*, *Csf3r*, *Gdf15*, *Mefv*, *Orm2*, *S100a9*, *Saa3*, and *Tnfrsf19* expression in PM_2.5_-exposed mice (Fig. 3D).

### ADSC transplantation attenuates pyroptosis in PM_2.5_-exposed lungs

**Fig. 4:**
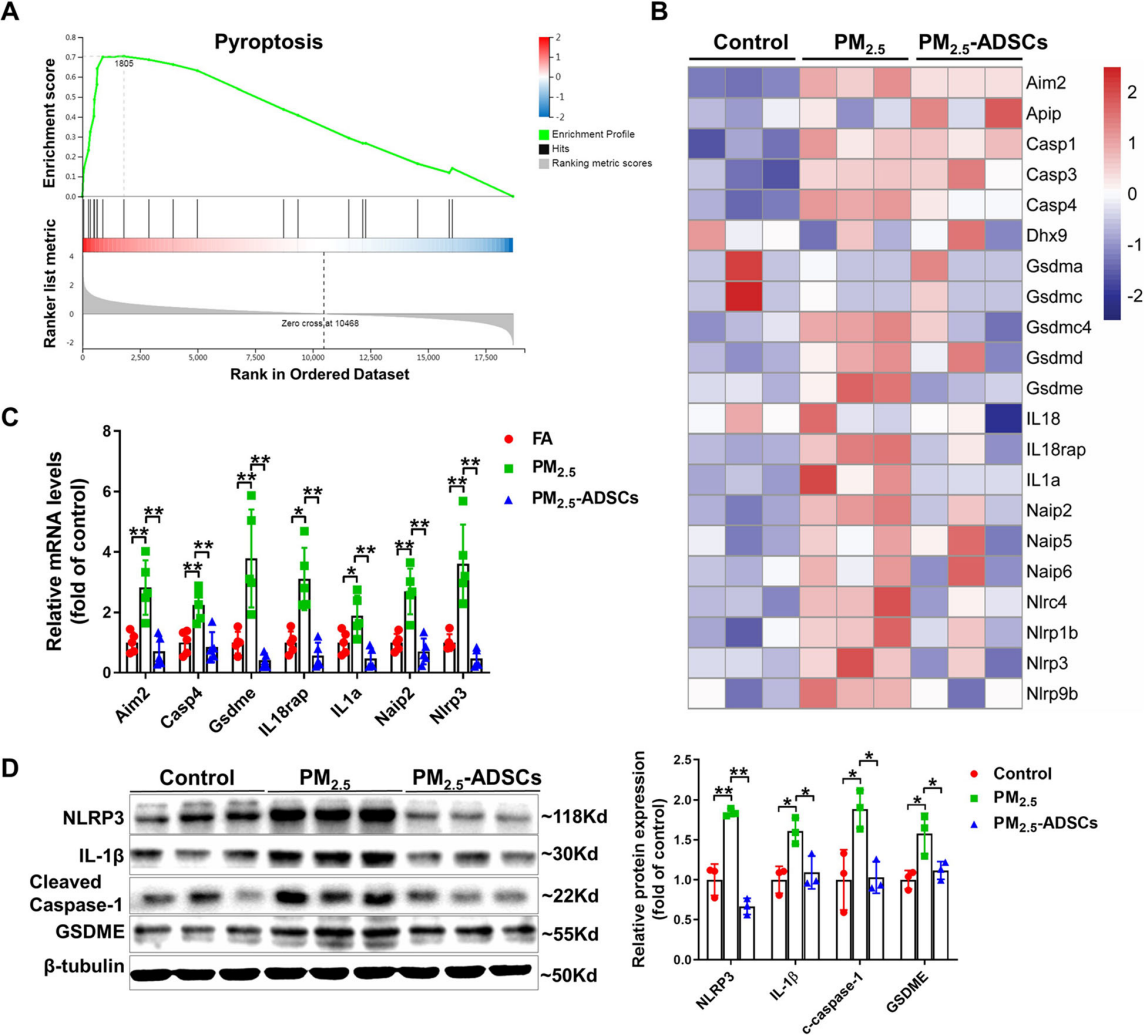
ADSC transplantation attenuated PM_2.5_-induced pyroptosis. **A** Gene set enrichment analysis plot showing the DEGs in the control group vs PM_2.5_ group enriched in the pyroptosis pathway. **B** Heat map showing the expression of the genes involved in the pyroptosis process. **C** The mRNA levels of pyroptotic genes were confirmed by qPCR. **D** Lysates of lung tissue were subjected to western blotting to determine the expression levels of NOD-like receptor protein 3 (NLRP3), IL-1β, cleaved caspase-1, and gasdermin E (GSDME). β-Tubulin was used as a loading control. In **C**, *N* = 5; in **D**, *N* = 3; the data are presented as the mean ± SD; asterisk indicates *p* < .05; double asterisks indicate *p* < 0.01

Recent studies have shown that PM_2.5_-induced pulmonary inflammation is associated with activation of NOD-like receptor protein 3 (NLRP3) and caspase-1 pathway, which can trigger cell pyroptosis [29]. GSEA results showed that the DEGs in the control group vs PM_2.5_ group were significantly enriched in inflammation and immune pathways, including the immune system process, adaptive/innate immune response, inflammatory response, chemotaxis, and immune response (Figure S3). Consistently, the DEGs in the control group vs PM_2.5_ group were also significantly enriched in the pyroptosis pathway (Fig. 4A). To further explore the mechanism underlying the activation of the pyroptosis pathway in PM_2.5_-exposed lungs, the expression profiles of genes involved in the pyroptosis pathway were visualized in the heat map (Fig. 4B), which demonstrated that the expression of most of the genes was upregulated in PM_2.5_-exposed lungs, while the upregulation of the expression of these genes was significantly attenuated by ADSC transplantation. The changes in gene expression were further validated by real-time qPCR, which confirmed that PM_2.5_-induced upregulation of pulmonary *Aim2*, *Casp4*, *Gsdme*, *IL18rap*, *IL1a*, *Naip2*, and *Nlrp3* gene expression was attenuated by ADSC transplantation (Fig. 4C). Western blotting also showed that PM_2.5_ exposure resulted in significant increases in the expression of NLRP3, IL-1β, cleaved caspase-1, and gasdermin E (GSDME), while the increase in the expression of these genes was significantly attenuated by ADSC treatment (Fig. 4D).

### ADSCs attenuate PM_2.5_-induced cardiac dysfunction and fibrosis

To determine whether ADSCs could alleviate cardiac dysfunction in PM_2.5_-exposed mice, we assessed the left ventricular (LV) function. Echocardiographic examination showed that PM_2.5_ exposure significantly decreased ejection fraction (EF) values, while this reduction was attenuated by ADSC treatment (Figure S4A-B). Furthermore, ADSCs also significantly attenuated PM_2.5_-induced upregulation of myocardial atrial natriuretic peptide (ANP; a marker for cardiac stress) (Figure S4C). As revealed by Masson’s staining and TUNEL staining, obvious fibrosis and cardiomyocyte apoptosis occurred in the hearts of the PM_2.5_-exposed mice, while the collagenous fibrotic area and apoptotic cell number in these hearts were significantly reduced by ADSC transplantation (Figure S4D-F). Western blotting showed that ADSC transplantation attenuated the PM_2.5_-induced decrease in Serca2a expression and increases in IL-1β, cleaved caspase-1, and GSDME expression in the heart (Figure S4G). Together, these results indicated that ADSCs effectively attenuates PM_2.5_-induced cardiac dysfunction in mice.

## Discussion

To the best of our knowledge, the present study provides the first direct evidence that ADSC transplantation is effective in attenuating PM_2.5_-induced lung injury and cardiac dysfunction. The mechanism underlying the protective effect of ADSCs was associated with the suppression of the inflammatory response and attenuation of cell death.

The finding that ADSC therapy decreased serum TNFα, IL-6, and IL-1β levels and pulmonary TNFα and IL-1β mRNA levels in PM_2.5_-exposed mice suggested that ADSCs protect against PM_2.5_-induced lung injury, at least partially, by suppressing the inflammatory response. The anti-inflammatory effects of ADSCs have also been observed in different pulmonary disease models, including models of bleomycin-induced interstitial pneumonia [30], lipopolysaccharide-induced pulmonary microvascular barrier damage [31], and ventilator-induced lung injury in rats [32]. Based on previous reports [33, 34], the molecular mechanism underlying the anti-inflammatory actions of ADSCs includes but is not limited to, secretion of immunosuppressive cytokines/EVs [35, 36], inhibition of microphage activation/infiltration [30, 37], a decrease in inflammatory cytokine production, and regulation of T-reg cells [38, 39]. Consistently, we demonstrated here that ADSCs had inhibitory effects on inflammatory cell infiltration and inflammatory pathway activation.

PM_2.5_ exposure can induce apoptosis in different cell lines and apoptosis induction mediated by PM_2.5_ contributes to its adverse health effects [16, 40]. Here, we found that the lung injury and cardiac dysfunction in PM_2.5_-exposed mice were associated with increases in the number of TUNEL-positive cells and the expression of cleaved caspase-3. It is notably that caspase 3 can also be enrolled in non-apoptotic processes [41]. The proapoptotic role of PM_2.5_ in the lungs was further confirmed by the decrease in Bcl-2/Bax ratio, which is an important inducer for mitochondrial cytochrome c release and caspase activation [42].

There is evidence that PM_2.5_ triggers pyroptosis by activating the NLRP3/caspase-1 signaling pathway in cells and lung tissues [29, 43]. In agreement with previous studies, we also observed that the mRNA expression of *Nlrp3*, *Aim2*, *Naip2*, and *I1a*, as well as protein expression of NLRP3, IL-1β, and cleaved caspase-1, were upregulated in PM_2.5_-exposed lungs. Pyroptosis can also be regulated by a caspase-1 independent pathway, in which caspase-4, caspase-5, and caspase-11 are apical activators [44]. Moreover, GSDME can induce a switch from caspase-3-mediated apoptosis induced by chemotherapy drugs to pyroptosis when it is highly expressed [45]. In the present study, we found that PM_2.5_ exposure increased the mRNA levels of *Casp4* and *Gsdme* and the protein expression of cleaved caspase-3 and GSDME in the lungs, indicating that PM_2.5_ induced pyroptosis via multiple mechanisms.

The finding that ADSC transplantation attenuated the increases in the number of TUNEL-positive cells, mRNA levels of pyroptosis-related genes and apoptosis- and pyroptosis-related proteins in the lungs and heart suggested that the protective effects of ADSCs against PM_2.5_-induced lung injury and cardiac dysfunction were associated with inhibition of cell apoptosis and pyroptosis. The antiapoptotic effect of ADSCs has also been observed in silicosis-exposed mice [46], heterotopic tracheal transplantation models [47], and radiation treated rats [48]. However, the antipyroptotic effect of ADSCs has not yet been reported. Considering that pyroptosis plays an important role in the pathogenesis of many respiratory and cardiovascular diseases, it is possible that ADSCs may have more potential clinical applications than expected.

It has been demonstrated that inflammatory chemokines provide axes for effective recruitment of therapeutic adult stem cells to home and engraft in the appropriate injured target tissues [49]. In the present study, we observed that the expression of chemokines, including *Ccl3/6*, *Cxcl1/2/3*, and *Cxcr1/2*, was upregulated in PM_2.5_-exposed lungs, which may result in a suitable inflammatory microenvironment for the recruitment of ADSCs. As previous studies have proven that ADSCs have the capacity to home and engraft in injured lungs [12, 13, 47], we did not label the ADSCs or track their homing and engraftment during the experimental process. Anyway, this is the limitation of our study. Another limitation of our study is we did not analysis the inflammatory cell infiltration with fluorescence activated cell sorting, which can reveal more information on inflammatory processes.

## Conclusion

In summary, our study indicates that ADSCs exhibit anti-inflammatory, anti-fibrotic, and anti-cell death effects in PM_2.5_-exposed lungs and that the underlying mechanism is associated with their immunosuppressive effects. Our results suggest that ADSC transplantation may be a potential therapeutic approach for air pollution-associated diseases.

## Supplementary Information

**Additional file 1: Table S1.** The quantitative real-time PCR primer information. **Table S2.** Detail information for antibodies. **Figure S1.** Flow cytometry analysis of adipose derived stem cells (ADSCs). ADSCs were stained with FITC-conjugated CD44, PE-conjugated CD73 and CD90.2, and eFluor® 450-conjugated CD34 antibodies for 30 min and then subjected to flow cytometry analysis. **Figure S2.** Morphology and differentiation potential of ADSCs. (A) Microscopic observation of the cultured ADSCs; (B) ADSCs were cultured in adipogenic differentiation induction medium for 14 days and then stained with oil red O; (C) ADSCs were cultured in osteogenic differentiation induction medium for 14 days and then stained with alizarin red. Scale bar = 200 μm. **Figure S3.** Gene set enrichment analysis shows the top 6 significant enriched pathways for DEGs of control vs PM_2.5_ group. **Figure S4.** ADSC transplantation attenuated PM_2.5_-induced cardiac dysfunction. (A, B) After PM_2.5_ exposure with or without ADSC transplantation, echocardiography was used to measure the left ventricular ejection fraction. (C) The mRNA levels of atrial natriuretic peptide (ANP) were measured. (D) Representative heart sections from control and PM_2.5_-exposed mice were stained with Masson’s trichrome stain (scale bar=100 μm) and a TUNEL assay kit (green) plus DAPI (blue) (scale bar=50 μm, the arrows point to TUNEL-positive cells). (E, F) The fibrotic area and TUNEL-positive cells were quantified. (G) Lysates of heart tissue were subjected to western blotting for Serca2, IL-1β, cleaved caspase-1 and GSDME. β-Tubulin was used as a loading control. In Figure A-N, *N* = 5; in Figure E-F, *N* = 6, in Figure G, *N* = 3; * indicates *p* < .05; ** indicates *p* < 0.01. **Figure S5.** Uncropped blot for Fig. 2. **Figure S6.** Uncropped blot for Fig. 4.

## Data Availability

The sequencing data for clean reads generated by this study have been deposited in the NCBI Sequence Read Archive (SRA) database (accession number PRJNA727861). All other data generated or analyzed during this study are included in this published article and its supplementary file.

## Abbreviations

3′-NT: 3′-Nitrotyrosine
4-HNE: 4-Hydroxynonenal
ADSC: Adipose-derived stem cell
ANP: Atrial natriuretic peptide
COPD: Chronic obstructive pulmonary disease
DEGs: Differentially expressed genes
EF: Ejection fraction
EV: Extracellular vesicle
FBS: Fetal bovine serum
GSDME: Gasdermin E
GSEA: Gene set enrichment analysis
H&E: Hematoxylin and eosin
IL: Interleukin
KEGG: Kyoto Encyclopedia of Genes and Genomes
LV: Left ventricular
NLRP3: NOD-like receptor protein 3
PM_2.5_: Fine particulate matter
PRDX: Peroxiredoxin
TGF-β: Transforming growth factor beta
TNFα: Tumor necrosis factor alpha
