# Supplementary material for: Adipose-derived stem cells therapy effectively attenuates PM2.5-induced lung injury
Source: Stem Cell Res Ther. 2021 Jun 19;12:355. doi: 10.1186/s13287-021-02441-3 (PMC8214780; doi:10.1186/s13287-021-02441-3)
Supplement: Supplementary file 1 — Additional file 1: Table S1. The quantitative real-time PCR primer information. Table S2. Detail information for antibodies. Figure S1. Flow cytometry analysis of adipose derived stem cells (ADSCs). ADSCs were stained with FITC-conjugated CD44, PE-conjugated CD73 and CD90.2, and eFluor® 450-conjugated CD34 antibodies for 30 min and then subjected to flow cytometry analysis. Figure S2. Morphology and differentiation potential of ADSCs. (A) Microscopic observation of the cultured ADSCs; (B) ADSCs were cultured in adipogenic differentiation induction medium for 14 days and then stained with oil red O; (C) ADSCs were cultured in osteogenic differentiation induction medium for 14 days and then stained with alizarin red. Scale bar = 200 μm. Figure S3. Gene set enrichment analysis shows the top 6 significant enriched pathways for DEGs of control vs PM2.5 group. Figure S4. ADSC transplantation attenuated PM2.5-induced cardiac dysfunction. (A, B) After PM2.5 exposure with or without ADSC transplantation, echocardiography was used to measure the left ventricular ejection fraction. (C) The mRNA levels of atrial natriuretic peptide (ANP) were measured. (D) Representative heart sections from control and PM2.5-exposed mice were stained with Masson’s trichrome stain (scale bar=100 μm) and a TUNEL assay kit (green) plus DAPI (blue) (scale bar=50 μm, the arrows point to TUNEL-positive cells). (E, F) The fibrotic area and TUNEL-positive cells were quantified. (G) Lysates of heart tissue were subjected to western blotting for Serca2, IL-1β, cleaved caspase-1 and GSDME. β-Tubulin was used as a loading control. In Figure A-N, N = 5; in Figure E-F, N = 6, in Figure G, N = 3; * indicates p < .05; ** indicates p < 0.01. Figure S5. Uncropped blot for Fig. 2. Figure S6. Uncropped blot for Fig. 4. [file 13287_2021_2441_MOESM1_ESM.doc]

**Supplemental Files**

**Adipose derived stem cells effectively attenuate PM_2.5_-induced lung injury**

Junling Gao^1#^, Juntao Yuan^1#^, Qun Liu^2#^, Yuanli Wang^1^, Huiwen Wang^2^, Yingjie Chen^3^, Wenjun Ding^1^, Guangju Ji^2*^, Zhongbing Lu^1*^

^1^College of Life Science, University of Chinese Academy of Sciences, Beijing, 100049, China

^2^Institute of Biophysics, Chinese Academy of Sciences, Beijing, 100101, China

^3^Department of Physiology and Biophysics, University of Mississippi Medical Center, Jackson, USA

# These authors contributed equally to this work

* Corresponding authors:

Guangju Ji, MD, PhD

Datun Road 15, Chaoyang district

Beijing 100101, China

Fax: 86-10-64849873; Tel: 86-10-64846720

[gj28@ibp.ac.cn](mailto:gj28@ibp.ac.cn)

Or

Zhongbing Lu, Ph.D,

19A Yuquanlu, Beijing, 100049, China

Tel & Fax: 86-10-69672630

E-mail: [luzhongbing@ucas.ac.cn](mailto:luzhongbing@ucas.ac.cn)

**Table S1. The quantitative real-time PCR primer information**

| Genes | Primers | Sequence( 5’-3’) |
| --- | --- | --- |
| *18s* | Forward | 5’-TTCTGGCCAACGGTCTAGACAAC-3’ |
|  | Reverse | 5’-CCAGTGGTCTTGGTGTGCTGA-3’ |
| *TNFα* | Forward | 5’- AGGGTCTGGGCCATAGAACT-3’ |
|  | Reverse | 5’- CCACCACGCTCTTCTGTCTAC -3’ |
| *IL-1β* | Forward | 5’- AGGTCAAAGGTTTGGAAGCA -3’ |
|  | Reverse | 5’- TGAAGCAGCTATGGCAACTG-3’ |
| *TGFβ* | Forward | 5’-CAACCCAGGTCCTTCCTAAA -3’ |
|  | Reverse | 5’-GGAGAGCCCTGGATACCAAC-3’ |
| *Collagen I* | Forward | 5’-TAGGCCATTGTGTATGCAGC-3’ |
|  | Reverse | 5'-ACATGTTCAGCTTTGTGGACC-3’ |
| *Collagen III* | Forward | 5’-TAGGACTGACCAAGGTGGCT-3’ |
|  | Reverse | 5’-GGAACCTGGTTTCTTCTCACC-3’ |
| *IL-6* | Forward | 5’-AACGATGATGCACTTGCAGA-3’ |
|  | Reverse | 5’-TGGTACTCCAGAAGACCAGAGG-3’ |
| *Orm2* | Forward | 5’-CAACATCACCATAGGCGACCC-3’ |
|  | Reverse | 5’-ATTTCCTGCCGGTAATCAGGG-3’ |
| *Ccl3* | Forward | 5’-TTCTCTGTACCATGACACTCTGC-3’ |
|  | Reverse | 5’-CGTGGAATCTTCCGGCTGTAG-3’ |
| *Ccl6* | Forward | 5’-GCTGGCCTCATACAAGAAATGG-3’ |
|  | Reverse | 5’-GCTTAGGCACCTCTGAACTCTC-3’ |
| *Saa3* | Forward | 5’-TGCCATCATTCTTTGCATCTTGA-3’ |
|  | Reverse | 5’-CCGTGAACTTCTGAACAGCCT-3’ |
| *Gdf15* | Forward | 5’-CTGGCAATGCCTGAACAACG-3’ |
|  | Reverse | 5’-GGTCGGGACTTGGTTCTGAG-3’ |
| *Csf3r* | Forward | 5’-CTGATCTTCTTGCTACTCCCCA-3’ |
|  | Reverse | 5’-GGTGTAGTTCAAGTGAGGCAG-3’ |
| *Tnfrsf9* | Forward | 5’-CGTGCAGAACTCCTGTGATAAC-3’ |
|  | Reverse | 5’-CGTGCAGAACTCCTGTGATAAC-3’ |
| *Mefv* | Forward | 5’-TCATCTGCTAAACACCCTGGA-3’ |
|  | Reverse | 5’-GGGATCTTAGAGTGGCCCTTC-3’ |
| *S100a9* | Forward | 5’-ATACTCTAGGAAGGAAGGACACC-3’ |
|  | Reverse | 5’-TCCATGATGTCATTTATGAGGGC-3’ |
| *Gsdme* | Forward | 5’-TGCAACTTCTAAGTCTGGTGACC-3’ |
|  | Reverse | 5’-CTCCACAACCACTGGACTGAG-3’ |
| *IL18rap* | Forward | 5’-CTGTGGACATATTCTGCAAGGG-3’ |
|  | Reverse | 5’-GCATGTACCACTGGACATCAGAT-3’ |
| *IL1a* | Forward | 5’-CGAAGACTACAGTTCTGCCATT-3’ |
|  | Reverse | 5’-GACGTTTCAGAGGTTCTCAGAG-3’ |
| *Casp4* | Forward | 5’-ACAAACACCCTGACAAACCAC-3’ |
|  | Reverse | 5’-CACTGCGTTCAGCATTGTTAAA-3’ |
| *Nlrp3* | Forward | 5’-ATTACCCGCCCGAGAAAGG-3’ |
|  | Reverse | 5’-TCGCAGCAAAGATCCACACAG-3’ |
| *Naip2* | Forward | 5’-AGCTTGGTGTCTGTTCTCTGT-3’ |
|  | Reverse | 5’-GCGGAAAGTAGCTTTGGTGTAG-3’ |
| *Aim2* | Forward | 5’-GTCACCAGTTCCTCAGTTGTG-3’ |
|  | Reverse | 5’-CACCTCCATTGTCCCTGTTTTAT-3’ |

**Table S2**. Detail information for antibodies

| **Provider** | **Targeted protein** | **Cat. no.** | **Clone number** | **Source** | **References** |
| --- | --- | --- | --- | --- | --- |
| **Cell Signaling Technology (**Danvers, MA, USA**)** | Bax | 2772 | N/A | rabbit | [1] |
|  | cleaved caspase-3 | 9664 | 5A1E | rabbit | [2] |
|  | cleaved caspase-1 | 89332 | E2G2I | rabbit | [3] |
| **Abcam PLC (Cambriage, UK)** | NLRP3 | ab263899 | EPR23094-1 | rabbit | [4] |
|  | IL-1β | ab200478 | 17H18L16 | rabbit | [5] |
|  | GSDME | ab215191 | EPR19859 | rabbit | [6] |
|  | SOD1 | ab183881 | N/A | rabbit | [7] |
|  | PRDX4 | ab184167 | EPR15458(B) | rabbit | [8] |
|  | Bcl-2 | ab194583 | N/A | rabbit | [9] |
|  | β-tubulin | ab6046 | N/A | rabbit | [9] |
| **Bioss Biotechnology Co. Ltd** | Neutrophil | bs-19701R | N/A | rabbit | [9] |
|  | Galectin 3 | bs-20700R | N/A | rabbit | [9] |
| **Zhongshan Company (Beijing, China)** | HRP-conjugated goat anti-rabbit antibody | #ZB-2301 | N/A | goat | [1] |

**
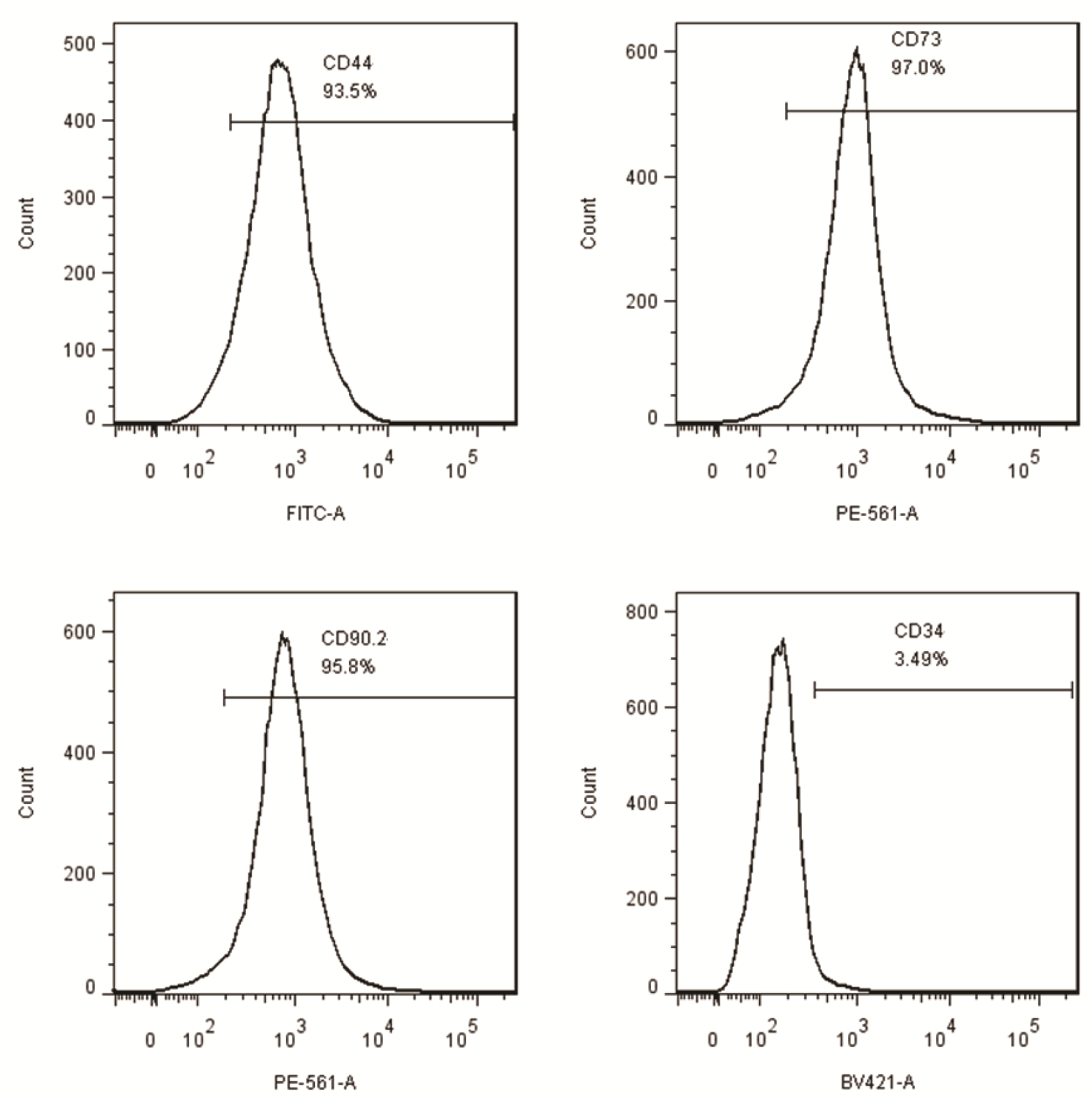
**

**Figure S1. Flow cytometry analysis of adipose derived stem cells (ADSCs).** ADSCs were stained with FITC-conjugated CD44, PE-conjugated CD73 and CD90.2, and eFluor® 450-conjugated CD34 antibodies for 30 min and then subjected to flow cytometry analysis.


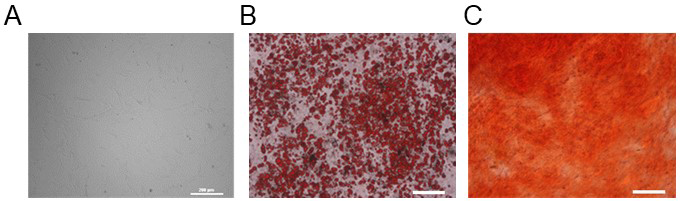


**Figure S2. Morphology and differentiation potential of ADSCs.** (A) Microscopic observation of the cultured ADSCs; (B) ADSCs were cultured in adipogenic differentiation induction medium for 14 days and then stained with oil red O; (C) ADSCs were cultured in osteogenic differentiation induction medium for 14 days and then stained with alizarin red. Scale bar = 200 µm.


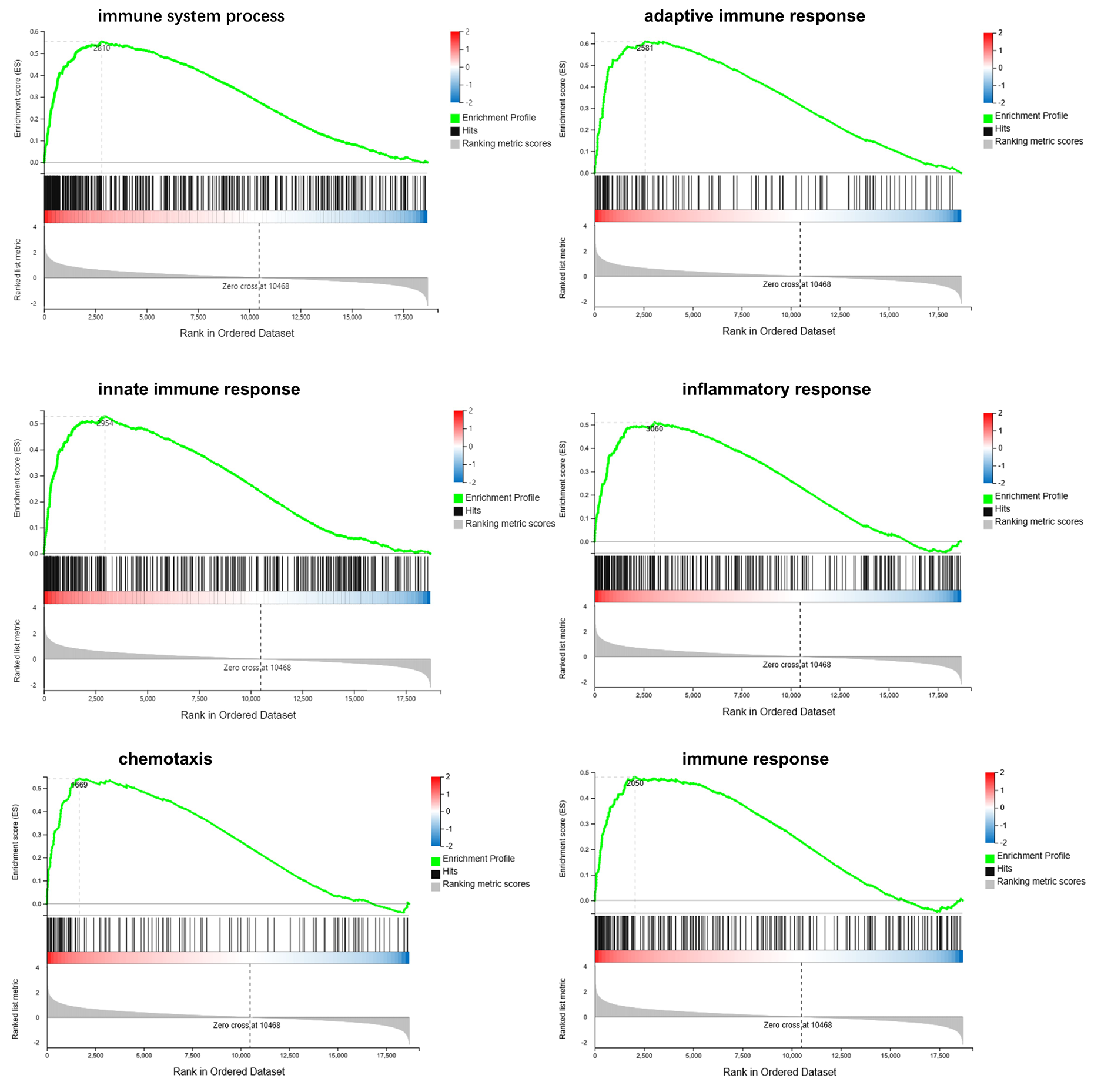


**Figure S3. Gene set enrichment analysis shows the top 6 significant enriched pathways for DEGs of control vs PM_2.5_ group.**


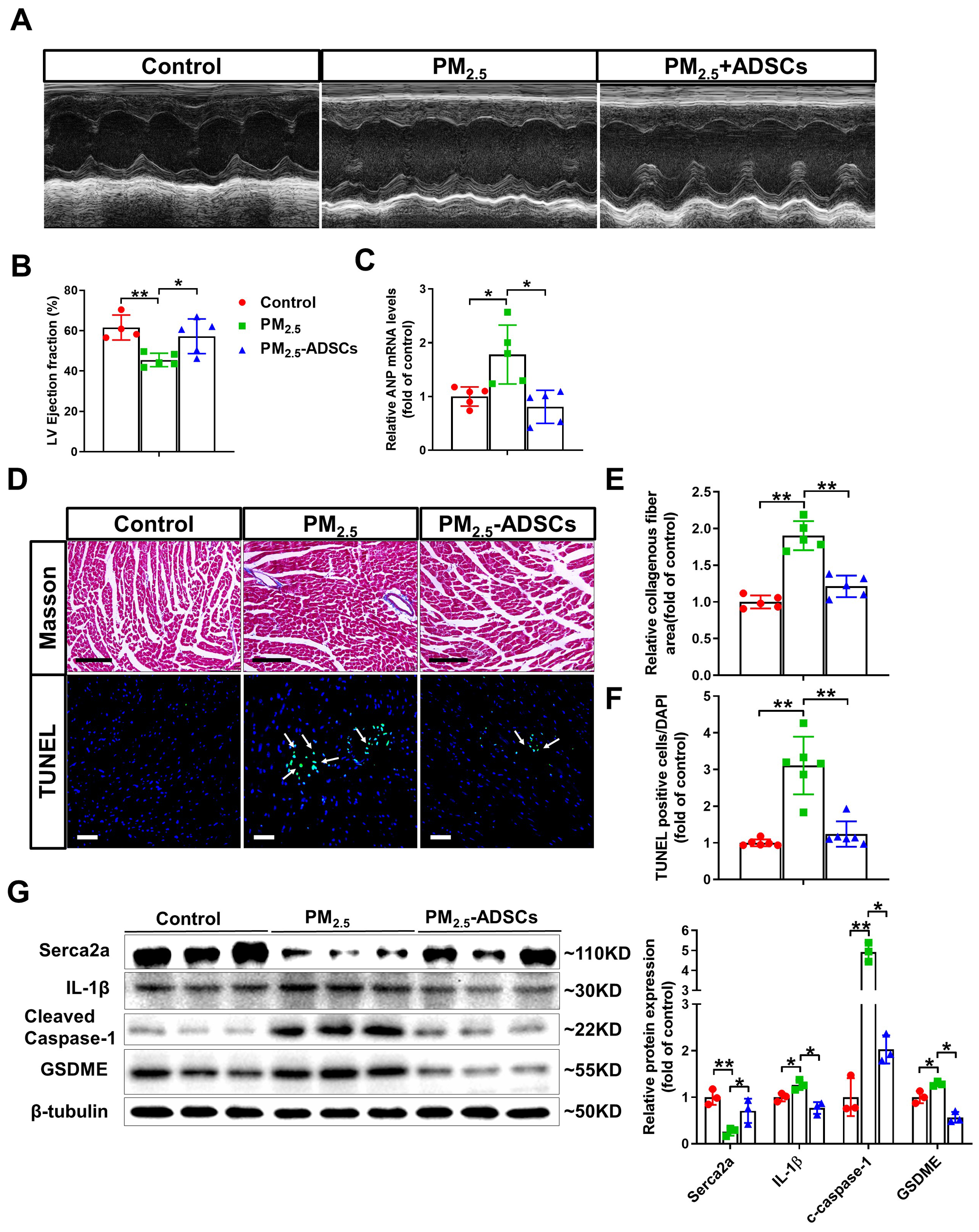


**Figure S4. ADSC transplantation attenuated PM_2.5_-induced cardiac dysfunction.** (A, B) After PM_2.5_ exposure with or without ADSC transplantation, echocardiography was used to measure the left ventricular ejection fraction. (C) The mRNA levels of atrial natriuretic peptide (ANP) were measured. (D) Representative heart sections from control and PM_2.5_-exposed mice were stained with Masson’s trichrome stain (scale bar=100 μm) and a TUNEL assay kit (green) plus DAPI (blue) (scale bar=50 μm, the arrows point to TUNEL-positive cells). (E, F) The fibrotic area and TUNEL-positive cells were quantified. (G) Lysates of heart tissue were subjected to western blotting for Serca2, IL-1β, cleaved caspase-1 and GSDME. β-Tubulin was used as a loading control. In Figure A-N, N=5; in Figure E-F, N=6, in Figure G, N=3; * indicates p<.05; ** indicates p<0.01.


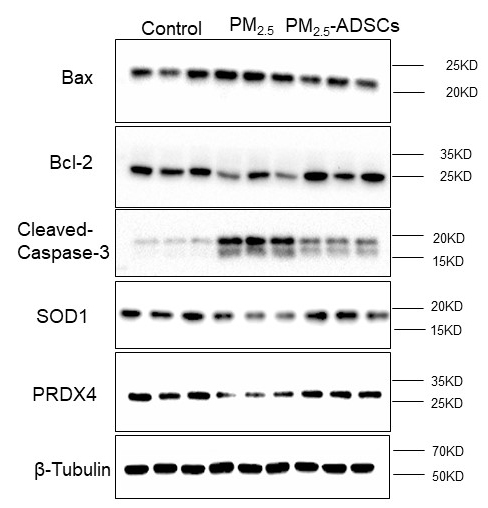


**Figure S5. Uncropped blot for Figure 2**


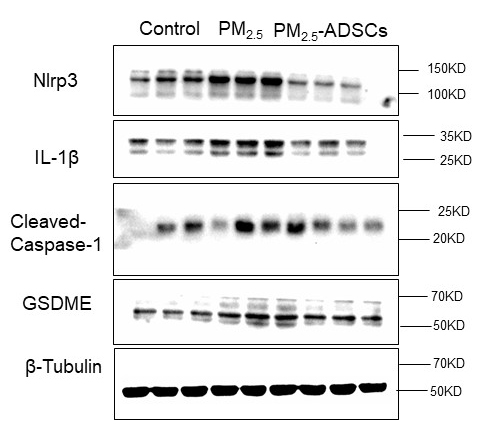


**Figure S6. Uncropped blot for Figure 4**

**References**

[1] Fan C, Feng J, Tang C, et al. Melatonin suppresses er stress-dependent proapoptotic effects via ampk in bone mesenchymal stem cells during mitochondrial oxidative damage. Stem cell research & therapy, 2020, 11: 442

[2] Fu J, Chen X, Liu X, et al. Elabela ameliorates hypoxic/ischemic-induced bone mesenchymal stem cell apoptosis via alleviation of mitochondrial dysfunction and activation of pi3k/akt and erk1/2 pathways. Stem cell research & therapy, 2020, 11: 541

[3] Mao Q, Liang XL, Zhang CL, et al. Lncrna klf3-as1 in human mesenchymal stem cell-derived exosomes ameliorates pyroptosis of cardiomyocytes and myocardial infarction through mir-138-5p/sirt1 axis. Stem cell research & therapy, 2019, 10: 393

[4] Yang Y, Liu C, Yang J, et al. Impairment of sirtuin 1-mediated DNA repair is involved in bisphenol a-induced aggravation of macrophage inflammation and atherosclerosis. Chemosphere, 2021, 265: 128997

[5] Wang X, Jiang L, Shi L, et al. Zearalenone induces nlrp3-dependent pyroptosis via activation of nf-κb modulated by autophagy in ins-1 cells. Toxicology, 2019, 428: 152304

[6] Wang K, Sun Q, Zhong X, et al. Structural mechanism for gsdmd targeting by autoprocessed caspases in pyroptosis. Cell, 2020, 180: 941-955.e920

[7] Maugeri G, D'Amico AG, Rasà DM, et al. Molecular mechanisms involved in the protective effect of pituitary adenylate cyclase-activating polypeptide in an in vitro model of amyotrophic lateral sclerosis. Journal of cellular physiology, 2019, 234: 5203-5214

[8] Lovatt M, Adnan K, Kocaba V, et al. Peroxiredoxin-1 regulates lipid peroxidation in corneal endothelial cells. Redox biology, 2020, 30: 101417

[9] Gao J, Yuan J, Wang Q, et al. Metformin protects against pm(2.5)-induced lung injury and cardiac dysfunction independent of amp-activated protein kinase α2. Redox biology, 2020, 28: 101345
